# Bariatric and Metabolic Surgery in Patients Older than 65 Years – a Multicenter Study

**DOI:** 10.1007/s11695-023-06750-9

**Published:** 2023-08-11

**Authors:** Natalia Dowgiałło-Gornowicz, Paweł Lech, Piotr Major, Collaborative Study Group, Paula Franczak, Paweł Jaworski, Klaudia Juszczuk, Izabela Karpińska, Bartosz Katkowski, Grzegorz Kowalski, Alicja Kucharska, Michał Orłowski, Monika Proczko-Stepaniak, Michał Szymański, Maciej Walędziak, Mateusz Wityk

**Affiliations:** 1 Department of General, Minimally Invasive and Elderly Surgery, Collegium Medicum, University of Warmia and Mazury, 10-045 Olsztyn, Poland; 2 2Nd Department of General Surgery, Jagiellonian University Medical College, 30-688 Cracow, Poland; 3 Department of General and Oncological Surgery, Ceynowa Hospital, Wejherowo, Poland; 4 Department of General, Oncological and Digestive Tract Surgery, Centre of Postgraduate Medical Education, Warsaw, Poland; 5 Department of General and Bariatric Surgery, Regional Specialist Hospital, Grudziądz, Poland; 6 Department of General and Vascular Surgery, Specialist Medical Center, Polanica Zdrój, Poland; 7 Surgery Clinic Mazan, Surgery Clinic Mazan, Katowice, Poland; 8 Department of General Surgery, Pro-Medica Hospital, Ełk, Poland; 9 Department of General, Endocrine and Transplant Surgery, Medical University of Gdansk, Gdańsk, Poland; 10 Department of General, Oncological, Metabolic and Thoracic Surgery, Military Institute of Medicine, Warsaw, Poland; 11 Department of General and Oncological Surgery, Voivodeship Specialist Hospital in Słupsk, Słupsk, Poland

**Keywords:** Metabolic surgery, Bariatric surgery, Older patients, Elderly, Type 2 diabetes, Hipertension

## Abstract

**Introduction:**

With the increase in life expectancy and a growing number of people suffering from obesity, bariatric and metabolic surgery is becoming a major concern in the elderly population. The study aimed to collect, systematize and present the available data on the surgical treatment of obesity among Polish patients over 65 years of age.

**Material and Methods:**

A retrospective study analysed patients over 65 years who underwent laparoscopic bariatric procedures in Poland from 2008 to 2022. The efficacy endpoints were percentage of excess weight loss (EWL%), percentage of total weight loss (%TWL), improvement in obesity-related diseases.

**Results:**

The group consisted of 284 patients (173 women, 60.9%). The mean follow-up was 47.5 months. The mean BMI before surgery was 43.1 kg/m2. 146 (51.4%) patients had T2D, and 244 (85.9%) had HT. The most common procedure was sleeve gastrectomy (82.0%). The mean EWL% after surgery was 50.9%, and the mean TWL% after surgery was 20.6%. There was the statistically significant difference between AGB vs OAGB, SG vs OAGB in %EWL (*p* = 0.0116, *p* = 0.009, respectively) and RYGB vs OAGB in %TWL (*p* = 0.0291). After surgery, 93 patients (63.7%) had complete or partial remission of T2D, and 112 patients (45.9%) had complete or partial remission of HT.

**Conclusion:**

Bariatric surgery appears to be a safe and effective method of treatment of obesity in patients over 65 years of age. OAGB seems to have better results in weight loss than SG, RYGB, and AGB in older patients.

**Graphical Abstract:**

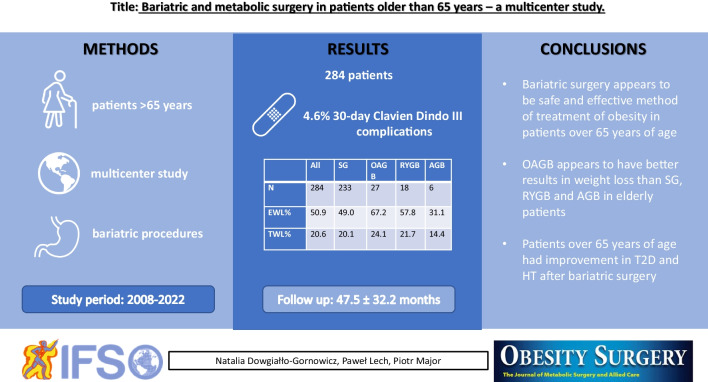

## Introduction

With increasing life expectancy and a growing number of people suffering from obesity, bariatric and metabolic surgery is becoming a major concern in the elderly population. [1, 2] In older patients, any surgery is a challenging procedure, as advanced age is associated with the development of diseases such as ischemic heart disease, respiratory failure, and frailty syndrome. [3] Combined with obesity-related diseases such as type 2 diabetes (T2D) or hypertension (HT), this is a serious problem for the multidisciplinary bariatric team.

There are several studies describing satisfactory results after bariatric surgery in older patients, compared to younger patients. [4, 5] The reporting of outcomes allowed the revision of guidelines, which now have no upper age limit. [6] However, the choice of the best procedure that will be less invasive for older patients while producing the best long-term results remains a matter of debate.

## Aim

The study aimed to collect, systematize and present available data on surgical treatment of obesity among Polish patients over 65 years of age. The primary outcomes were weight loss and improvement in obesity-related diseases. The secondary outcome was the safety of the surgery.

## Material and Methods

It is a multicenter, retrospective analysis of a collected database of patients undergoing laparoscopic bariatric procedures in Poland from 2008 to 2022. The Metabolic and Bariatric Surgery Chapter and the Videosurgery Chapter of the Association of Polish Surgeons took patronage of the study. Inclusion criteria for this study were meeting the eligibility criteria for bariatric surgery and being over 65 years of age. [7] There were 364 patients who meet the criteria. Patients with missing or inconsistent data were excluded from the study. The follow up rate was 78.0%.

The database contained demographic characteristics of patients (sex, age, maximal weight, weight before the surgery, body mass index) and information on obesity-related diseases: T2D, HT, dyslipidemia, obstructive sleep apnea, osteoarthritis and other comorbidities. It also included information on the surgery (type of surgery, duration of surgery, length of hospital stay, complications), and outcomes of bariatric treatment (current weight and BMI, obesity-related diseases remission). The outcomes of bariatric surgery were described according to the standardized outcomes reporting. [8] Complete remission of T2D is normal measures of glucose metabolism (HbA1c < 6%, fasting blood glucose (FBG) < 100 mg/dL) in the absence of antidiabetic medications. Partial remission of T2D is subdiabetic hyperglycemia (HbA1c 6%–6.4%, FBG 100–125 mg/dL) in the absence of antidiabetic medications. And improvement in T2D is statistically significant reduction in HbA1c and FBG not meeting criteria for remission or decrease in antidiabetic medications requirement (by discontinuing insulin or 1 oral agent, or ½ reduction in dose) [8]. Complete remission of HT is being normotensive (blood pressure (BP) < 120/80) off antihypertensive medication. Partial remission of HT is defined as prehypertension values (BP 120–140/80–89) when off medication. Improvement in HT is defined as a decrease in dosage or number of antihypertensive medication or decrease in systolic or diastolic BP on the same medication [8]. All results correspond to the follow-up time.

Surgical techniques and perioperative care protocols were standard at each participating center. Patients were treated by a multidisciplinary team of surgeons, physicians, nurses, nutritionists, and psychologists at each bariatric center. There was no specific pathway for bariatric patients over the age of 65. All patients follow the ERASB protocol. [9] Every patient prepared for surgery underwent echocardiography, electrocardiography, gastroscopy, chest x-ray, abdominal ultrasound and necessary laboratory tests. Depending on the results, patients are consulted by a specialist. All operations were performed in accordance with the guidelines. [10] Sleeve gastrectomy was performed using bougie size of 36F, starting 4–6 cm from the pylorus. The length of the biliopancreatic limb was approximately 200 cm from the ligament of Treitz in the OAGB. The biliopancreatic limb length was approximately 100 cm and Roux-en-Y limb length was approximately 150 cm in RYGB.

### Statistical Analysis

A descriptive statistical analysis was conducted. All data were analysed using Statistica software 13.PL (StatSoft Inc.). The normal distribution was checked using the Shapiro–Wilk test. A number and a percentage were used for categorical variables. For continuous variables with normal distribution, the mean and standard deviation were used. Student’s t-test was applied for the independent variables. A p-value of < 0.05 was considered statistically significant.

## Results

### Patient Characteristics and Indications

The group consisted of 284 patients (173 women, 60.9%). The mean age was 66.7 years ± 1.6 years. The mean follow-up was 47.5 months ± 32.2 months. The mean BMI before surgery was 43.1 kg/m2 ± 5.8 kg/m2. A total of 146 (51.4%) patients suffered from T2D,244 (85.9%) from HT and 94 (33.1%) dyslipidemia, Table 1. Other comorbidities are presented in Table 1. Specific indications for bariatric procedures were too high doses of drugs (10.6%), unregulated T2D (6.7%) or HT (3.5%), orthopedic (8.5%), cardiological (0.7%) or neurosurgical (0.7%) indications, and gastrointestinal reflux disease (0.4%).

**Table 1:** Characteristics of patients. (SD – standard deviation)

Variable	Value
Female, sex, no (%)	173 (60.9)
Age, mean ± SD (range) [years]	66.7 ± 1.6 (65–74)
BMI, mean ± SD (range) [kg/m^2^]	43.1 ± 5.8 (26.9–65.6)
Follow up, mean ± SD (range) [months]	47.5 ± 32.2 (1–172)
Comorbidities, no, (%)	
Type 2 diabetes	146 (51.4)
Hypertension	244 (85.9)
Dyslipidemia	94 (33.1)
Ischemic heart disease	33 (11.6)
History of heart failure	10 (3.5)
Atrial fibrillation/Other arrhytmias	16 (5.6) / 5 (1.8)
Obstructive sleep apnea	22 (7.7)
Chronic obstructive pulmonary disease	12 (4.2)
Asthma	16 (5.6)
Sarcoidosis	1 (0.4)
Hyperuricemia	31 (10.9)
Rheumatoid arthritis	5 (1.8)
Osteoarthritis	34 (12.0)
Depression/Anxiety disorders	6 (2.1) / 5 (1.8)
Hypothyroidism	30 (10.6)
Hyperthyroidism	1 (0.4)
Glaucoma	2 (0.7)
Cataract	2 (0.7)
Urinary incontinence	11 (3.9)
Prostate hyperplasia	5 (1.8)
Chronic kidney failure	4 (1.4)
Lower limb varicose vein	11 (3.9)
Collitis ulcerosa	1 (0.4)

### Type of Surgeries

There were 233 (82.0%) sleeve gastrectomies (SG), 27 (9.6%) one anastomosis gastric bypasses (OAGB), 18 (6.3%) Roux-en-Y gastric bypasses (RYGB), and 6 (2.1%) adjustable gastric bands (AGB), Table 2. The mean operative time was 68.7 ± 29.9 min for SG, 145.2 ± 38.6 min for RYGB, 91.0 ± 36.2 min for OAGB, and 85.0 ± 11.2 min for AGB.

**Table 2:** Outcomes of surgery

	All	SG	OAGB	RYGB	AGB
N	284	233 (82.0%)	27 (9.6%)	18 (6.3%)	6 (2.1%)
Operative time [minutes]	76.0 ± 38.0	68.7 ± 29.9	91.0 ± 36.2	145.2 ± 38.6	85 ± 11.2
Lenght of hospital stay [days]	2.54 ± 2.5	2.38 ± 1.6	2.0 ± 1.5	5.5 ± 7.3	2.5 ± 1.1
BMI preop. [kg/m2]	43.1 ± 5.8	43.7 ± 5.5	40.0 ± 5.4	40.7 ± 7.4	40.9 ± 5.1
BMI postop. [kg/m2]	34.1 ± 5.5	34.7 ± 5.4	30.0 ± 3.9	31.3 ± 4.7	34.4 ± 4.2
EWL%	50.9 ± 25.5	49.0 ± 23.7	67.2 ± 26.0	57.8 ± 28.9	31.1 ± 39.5
TWL%	20.6 ± 10.0	20.1 ± 9.4	24.1 ± 10.9	21.7 ± 11.9	14.4 ± 15.7

The observation period was divided into the years 2007–2016 and 2017–2022. In the earlier period, There were 77.2% SG, 14.0% OAGB, 3.5% RYGB, 5.3% AGB. Later, there were 83.3% SG, 8.4% OAGB, 7.0% RYGB, and 1.3% AGB.

### Weight Loss

The mean EWL% after surgery was 50.9% ± 25.5. It was 49.0% for SG, 67.2% for OAGB, 57.8% for RYGB, and 31.1% for AGB, Table 2. The mean TWL% after surgery was 20.6% ± 10.0. It was 20.1% for SG, 24.1% for OAGB, 21.7% for RYGB, and 14.4% for AGB, Table 2. There was a statistically significant difference between AGB vs OAGB, SG vs OAGB in %EWL (p = 0.0116, p = 0.009 respectively) and RYGB vs OAGB in %TWL (p = 0.0291), Table 3.

**Table 3:** Comparision of procedures

Procedure	P value of %EWL	P value of %TWL
AGB VS OAGB	0,0116	0,0899
AGB VS RYGB	0,1027	0,4788
AGB VS SG	0,0757	0,1387
OAGB VS SG	0,0009	0,0593
OAGB VS RYGB	0,2709	0,0291
RYGB VS SG	0,1407	0,2261

### Improvement in Obesity-Related Diseases

After surgery, 93 patients (63.7%) had complete or partial remission of T2D and, 112 patients (45.9%) had complete or partial remission of HT. Only 10 patients (6.8%) with T2D and 34 patients (13.9%) with HT showed no changes in the treatment of obesity-related diseases after surgery, Table 4.

**Table 4:** Changes in obesity-related diseases

	All	SG	OAGB	RYGB	AGB
T2D					
Remission, no, (%)	51 (34.9)	35 (30.7)	9 (60)	6 (42.9)	1 (33.3)
Partial remission, no, (%)	42 (28.8)	35 (30.7)	3 (15)	2 (11.1)	2 (66.7)
Improvement, no, (%)	43 (29.5)	34 (29.8)	3 (15)	6 (42.9)	-
No changes, no, (%)	10 (6.8)	10 (8.8)	-	-	-
HT					
Remission, no, (%)	55 (22.5)	46 (23.1)	7 (30.4)	2 (11.1)	-
Partial remission, no, (%)	57 (23.4)	45 (22.6)	2 (8.7)	6 (33.3)	4 (100)
Improvement, no, (%)	98 (40.2)	82 (41.2)	11 (47.8)	5 (27.8)	-
No changes, no, (%)	34 (13.9)	26 (13.1)	3 (13.1)	5 (27.8)	-

### Complications and Length of Stay

There were 20 (7.0%) complications in the analysed group. There were 13 (4.6%) 30-day Clavien Dindo III complications: 8 (2.8%) intraperitoneal bleedings, 3 (1.1%) leaks, and.

2 intraabdominal abscesses (0.7%). Five of the eight patients with intraperitoneal bleeding had hypertension. None of these patients received antiplatelet therapy. There were 3 (1.1%) tightening of sleeves requiring reoperation, 2 (0.7%) bile refluxes, 1 (0.4%) mesenteric vein thrombosis, and 1 (0.4%) internal hernia. The tightening of sleeves occurred in 3 different centers, with no correlation with oversewing the staple line. There was no postoperative death. The mean length of hospital stay was 2.5 ± 6.7 days.

## Discussion

Our study is a retrospective analysis of 284 patients over the age of 65 who underwent bariatric and metabolic surgery. This study included data on the largest group of patients over 65 years of age collected as a part of a multidisciplinary long-term follow-up reporting project in Poland. The study showed that bariatric surgery is performed on older patients. The oldest at the time of surgery was 74 years old.

In our study, the mean follow-up was 47.5 months, and %EWL was 50.9%. There are few studies describing such a long follow-up of patients older than 65 years. [11–13] The mean %EWL after 48 months was approximately 42% after SG, 66% after RYGB, and 70% after OAGB. [11–13] This is comparable to our results. The results of our study may indicate that OAGB is more effective in patients older than 65 years than SG and RYGB.

SG has become the most frequently performed procedure not only in our country, but also globally. [14, 15] Our study shows that the choice of procedure is more favorable for the SG than other procedures over the entire period. Trends changed along with the change of nationwide trends. [15] However, our findings indicate that surgeons more frequently opted for SG and OAGB procedures in patients aged 65 years and above, while RYGB was chosen less frequently [14, 15] An analysis by Bhandari et al. showed that 50% of patients over 65 years old underwent SG, while 28.8% underwent RYGB in years 2010–2013, which is in line with the prevailing trends in that region at the time. [12, 16] In our part of Europe, SG is performed much more often than in other parts of the world, which is also associated with the preference of performing these procedures in elderly people. However, the limited available data on the decision-making process for selecting procedures in elderly patients highlights the need for further research in this area.

A total of 93.2% of patients had at least improvement in T2D, 63.7% had complete or partial remission of T2D. A total of 86.1% had at least improvement in HT and 45.9% had complete or partial remission of HT. This is in line with other studies. [4, 12]. Bhandari et al. reported a non-significant comparison between older and younger patients in improvement in obesity-related comorbidities. [12]

Recent studies showed that elderly patients have a higher risk of complications than younger patients, but it is still considered safe. [17–20] On the other hand, Quirante et al. described that older patients are initially in worse condition than younger patients, so the overall risk of adverse events appears comparable. [21] The largest data from National Readmission Database from the United States described by Mabeza et al. showed that patients older than 65 years experience significantly higher mortality, morbidity, and resource consumption following bariatric surgery compared to younger adults. [22] In our study, there were 7.0% complications. 13 (4.6%) were 30-day Clavien Dindo III complications requiring reoperation. The leak rate was 1.1% in our study. A large MBSAQIP Analysis by Alizadeh et al. showed that the overall leak rate for bariatric surgery is 0.7% and the risk is not related to age. [23] The bleeding rate after bariatric surgery ranged from 1 to 6%, in our paper the bleeding rate was 2.8%. [24, 25] It seems that bariatric and metabolic surgery can be assessed as safe in patients over 65 years of age.

Moreover recent studies have shown that it is the presence of complications of old age, including frailty syndrome and diseases associated with old age, that causes an increase in the number of complications, not age itself. [26, 27] There are several studies on safety of bariatric surgery in patients over 70 years of age [28, 29]. Parmar et al. compared patients over the age of 70 with those under the age of 60. [28] There were no significant differences in treatment outcomes or complications. A recent analysis by Hansel et al. showed that selected patients over 69 should be offered bariatric surgery. [29] It may prolong the life even in septuagenarian patients. Therefore, when qualifying a patient for bariatric and metabolic surgery, one should take into account not so much the chronological age but the biological age and the consequences that follow.

The limitation of the study is its retrospective nature. Second, the majority of surgeries were SG, so the results of other procedures should be interpreted with caution due to the small sample size. We do not have partial data on outcomes. The obtained results are the endpoint of the follow-up. Moreover, we did not measure the quality of life of these patients. However, due to the small number of long-term follow-up studies, our study seems to have relevance in bariatric surgery.

## Conclusions

Bariatric surgery appears to be a safe and effective method of treatment of obesity in patients over 65 years of age. OAGB appears to have better weight loss results than SG, RYGB, and AGB in elderly patients. Patients over 65 years of age had improvement in T2D and HT after bariatric surgery.
